# Zika virus infection in a pregnant Canadian traveler with congenital fetal malformations noted by ultrasonography at 14-weeks gestation

**DOI:** 10.1186/s40794-018-0062-8

**Published:** 2018-04-04

**Authors:** Kevin L. Schwartz, Tiffany Chan, Nanky Rai, Kellie E. Murphy, Wendy Whittle, Michael A. Drebot, Jonathan Gubbay, Andrea K. Boggild

**Affiliations:** 1 Public Health Ontario, Toronto, Canada; 2 St. Joseph’s Health Sciences Centre, Toronto, Canada; 3 Dalla Lana School of Public Health, University of Toronto, Toronto, Canada; 4 Division of Infectious Diseases, Department of Medicine, University of Toronto, Toronto, Canada; 5 Department of Family and Community Medicine, University of Toronto, Toronto, Canada; 6 Department of Obstetrics and Gynecology, University of Toronto, Toronto, Canada; 7 Sinai Health System, Toronto, Canada; 8 National Microbiology Laboratory, Public Health Agency of Canada, Winnipeg, Canada; 9 Department of Laboratory Medicine and Pathobiology, University of Toronto, Toronto, Canada; 10 Tropical Disease Unit, Toronto General Hospital, 200 Elizabeth Street, 13EN-218, Toronto, ON M5G 2C4 Canada

**Keywords:** Arbovirus, Congenital infections, Fetal sonography, Pregnancy and travel, Zika virus

## Abstract

**Background:**

Following emergence of Zika virus in the Americas, a devastating new congenital syndrome has been documented, leading to significant morbidity among Zika-infected fetuses and neonates.

**Case presentation:**

A 29-year-old pregnant woman infected with Zika virus at 9-weeks gestation in Trinidad presented with one-month of fever, headache, and myalgia with persistent viremia. Significant fetal abnormalities were identified at 14-week ultrasound, which is the earliest ultrasound to describe a severely affected fetus following Zika virus infection to our knowledge.

**Conclusions:**

We discuss the implications of prolonged maternal viremia and the spectrum of congenital Zika syndrome detectable by fetal ultrasound.

## Background

Following reports of associations between congenital microcephaly and Zika virus (ZIKV) infection in pregnancy, the World Health Organization (WHO) declared ZIKV a Public Health Emergency of International Concern. ZIKV was first described over 70 years ago with limited spread outside Africa and no description of congenital abnormalities. There had been no newly discovered congenital infectious disease since cytomegalovirus and rubella in the mid-twentieth century [1, 2].

ZIKV is primarily transmitted through the bite of the day biting *Aedes* species of mosquito. Symptoms most commonly associated with ZIKV infection include diffuse or focal rash, conjunctivitis, fever, myalgia, and arthralgia [3]. Approximately 80% of ZIKV infections are asymptomatic [4].

Identification of this emerging arboviral congenital infection has led to justifiable concern among patients, physicians, and public health officials. In the absence of a licensed vaccine and specific treatment, the focus has been on prevention and calls for enhanced understanding of the epidemic. We report a case of a pregnant woman infected with ZIKV during the first trimester with prolonged viremia and significant fetal neurological compromise identified at 14-weeks gestation.

## Case presentation

A 29-year-old pregnant woman from Canada, gravida 4 para 3, developed a low-grade fever and a generalized rash at 9-weeks gestation while visiting friends and relatives in Trinidad. Symptom onset occurred 6-weeks after arriving in Trinidad and all symptoms lasted for 4 days, followed by approximately one month of fever, myalgia, and retro-orbital headaches. Seven days after symptom onset she sought medical care in Trinidad with her 4-year-old child who was experiencing similar symptoms. Her blood tested negative in Trinidad by real-time reverse transcription polymerase chain reaction (PCR) for dengue and chikungunya viruses, but was positive for ZIKV. An ultrasonographic examination was not performed locally in Trinidad. One month after symptom onset she returned to Canada for further evaluation.

The patient was evaluated in our centre at 13 weeks 6 days gestation, which corresponded to one month post-symptom onset. Past medical history was significant for mild cerebral palsy and scoliosis. Her only medication was a prenatal multivitamin. She denied substance use during pregnancy. On examination, temperature was 38.1 °C. Heart rate and blood pressure were normal. There was no conjunctivitis, lymphadenopathy, or organomegaly. Cardiorespiratory exam was normal. The previously mentioned diffuse rash had resolved.

Blood was drawn, and at 30 days post-symptom onset, ZIKV PCR, performed by Public Health Ontario Laboratory (PHOL), was positive at a cycle threshold (CT) of 31.85 using a commercial assay [RealStar® Zika Virus RT-PCR Kit (Altona Diagnostics, Hamburg, Germany). Urine ZIKV PCR was negative by the same assay. Dengue IgM and IgG were reactive, and chikungunya IgM and IgG were non-reactive. ZIKV IgM ELISA and plaque reduction neutralization test (PRNT) serology was performed at Canada’s National Microbiology Laboratory (NML) as previously described [5]. The ZIKV IgM ELISA was positive, however, the PRNT was inconclusive. Similar neutralization titres were demonstrated for both ZIKV and dengue virus (ZIKV titer 320, dengue virus titer 160) and were consistent with a previous exposure to a non-ZIKV flavivirus.

**Fig. 1:**
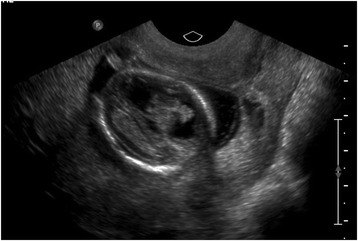
Asymmetrical cerebral ventricles. Dilated right ventricle with prominent, irregular choroid plexus

Fetal ultrasound performed at 14-weeks gestation revealed a single live intrauterine pregnancy with bilateral fluid collections in the fetal neck likely representing dilated jugular lymph sacs. The larger collection was 6-mm in size. Abnormal fetal intracranial anatomy was identified. There was asymmetry in the appearance of the lateral cerebral ventricles (Figure 1), the right ventricle appeared dilated especially in the frontal horn, and the left ventricle appeared small. The left choroid plexus was prominent in the frontal horn region and the third ventricle appeared slightly dilated. Head circumference was 14.5 weeks by size. No intracranial calcifications were seen.

Therapeutic termination of the pregnancy was performed by dilation and curettage at 15-weeks gestation. Initial molecular testing of fetal tissue was performed at PHOL. ZIKV RNA was detected in the umbilical cord (CT 31.70), placenta (CT 24.89), liver (CT 24.62), and fetal neural tissue (CT 17.42) using the RealStar® Zika Virus RT-PCR Kit. In addition, ZIKV sequence was confirmed in all fetal samples by RT-PCR and Sanger amplicon sequencing targeting 192 nucleotides of the ZIKV NS5 gene, based on a previously published protocol [6]. All fetal tissue specimens were forwarded to NML and retested using the CDC-designed real-time RT-PCR assay based on the ZIKV strain responsible for the 2007 outbreak in Micronesia, targeting the ZIKV envelope (*E*) and premembrane (*prM*) genes [5]. ZIKV RNA was again detected in umbilical cord, placenta, and fetal neural tissue specimens. Transmission electron microscopy was performed at PHOL on placenta, umbilical cord, and neural tissue specimens, however no viral particles were visualized. Fetal tissue karyotyping for aneuploidy was performed as per standard practice, and the result was a normal male chromosomal microarray pattern.

## Discussion

Uncertainties remain surrounding the risk of congenital Zika syndrome following maternal infection as well as the spectrum of disease. A recent report found that the proportion of birth defects among 442 pregnancies with laboratory evidence of ZIKV infection [7] was 20-times higher compared to the prevalence from three population-based birth defect surveillance programs during the 2013–2014 pre-Zika years [8]. Brain abnormalities or microcephaly were the most frequently recorded anomalies [8], but a number of case reports and case series have since emerged describing the spectrum of fetal effects of maternal Zika virus infection [9, 10]. We present a case of first trimester maternal infection with significant fetal neurological malformations identified by a 14-week ultrasound following congenital infection at 9-weeks gestation. To our knowledge this is the earliest ultrasound to describe a severely affected fetus following Zika virus infection.

Previous reports have described microcephaly, intracranial calcifications, and other abnormalities in the 2nd and 3rd trimesters (Table 1) [11–18]. The greatest risk for microcephaly has been reported to occur with ZIKV infection during the first trimester of pregnancy (Table 1), with estimated risks of possible Zika-associated birth defects in the first, second, and third trimesters of 8%, 5%, and 4%, respectively [19]. However, severely affected fetuses have been described after normal 2nd trimester ultrasounds, with subsequent abnormalities detected in the 3rd trimester (Table 1). Interestingly, there have also been reports of no differences in head circumference among infants born to women with possible ZIKV infection during pregnancy compared to women without infection [20]. The case presented herein describing severe neurological malformation in the early 2nd trimester contributes to our understanding of the spectrum of fetal abnormalities from maternal ZIKV infection.

**Table 1:** Summary of previously reported Zika congenital syndrome fetal ultrasound abnormalities

Gestation at First Abnormal Ultrasound (weeks)	Study Author, Year	Country of Infection	Gestation at Infection	Ultrasound Findings	Outcome
18	Sarno et al., 2016 [11]	Brazil	NR	Microcephaly, hydranencephaly, intracranial calcifications, destructive lesions of posterior fossa. Hydrothorax, ascites, subcutaneous edema	Fetal demise at 32 weeks
19	Driggers et al., 2016 [12]	Unknown (Guatemala, Mexico or Belize)	11 weeks	Possible intraventricular haemorrhage, corpus callosum agenesis	TA at 21 weeks
19	Pomar et al., 2017 [13]	French Guiana	First trimester	Severe microcephaly, cerebral hypoplasia	Delivery at 32 weeks
19.3	Cortes et al., 2018 [14]	Colombia	NR	Enlarged subarachnoid space, decreased brain volume, ventriculomegaly, malformations in cortical development, simplified gyral pattern, hypoplasic corpus callosum	Unknown
20	Pomar et al., 2017 [13]	French Guiana	First trimester	Cystic dilation of the cisterna magna, cortical hyperechogenicity, vermian hypoplasia, pericallosal echogenicity	Delivery at 38 weeks
21	Besnard et al., 2016 [15]	French Polynesia	First trimester	Microcephaly, parenchymal calcifications, ventriculomegaly, absent corpus callosum, absent cavum septi pellucidi	TA at unknown weeks
22	Pomar et al., 2017 [13]	French Guiana	First trimester	Periventricular hyperechogenicity, irregular ventricular walls, parenchymal and periventricular calcifications	Delivery at 41 weeks
22	Carvalho et al., 2016 [16]	Brazil	9 weeks	Microcephaly, parenchymal and ocular calcifications, Dandy Walker malformation, arthrogryposis	Fetal demise, CS at 37 weeks
22	Besnard et al., 2016 [15]	French Polynesia	First trimester	Microcephaly, parenchymal calcifications, ventriculomegaly, absent corpus callosum, absent of cavum septi pellucidi, enlarged pericerebral space	TA at unknown weeks
24	Carvalho et al., 2016 [16]	Brazil	5 weeks	Dandy Walker malformation, fetal growth restriction, arthrogryposis.	Fetal demise, CS 38.3 weeks
25.3	Cortes et al., 2018 [14]	Colombia	NR	Enlarged subarachnoid space, decreased brain volume, ventriculomegaly, malformations in cortical development, simplified gyral pattern, hypoplasic corpus callosum	NR
26.5	Cortes et al., 2018 [14]	Colombia	NR	Dandy Walker cerebella malformation	NR
27	Carvalho et al., 2016 [16]	Brazil	11 weeks	Microcephaly, ventriculomegaly bilaterally, parenchymal calcifications, increased subarachnoid space	CS delivery of live infant at 38 weeks
27	Cortes et al., 2018 [14]	Colombia	NR	Decreased brain volume, ventriculomegaly, simplified gyral pattern, hypoplastic corpus callosum	NR
28	Pomar et al., 2017 [13]	French Guiana	Second trimester	Severe ventriculomegaly with irregular ventricular walls, thin hypoplastic corpus callosum	TA at 29 weeks
28	Carvalho et al., 2016 [16]	Brazil	8 weeks	Microcephaly, ventriculomegaly, parenchymal calcification, and oligohydraminios	SVD of live infant at 39 weeks
28	Carvalho et al., 2016 [16]	Brazil	8 weeks	Microcephaly, ventriculomegaly, parenchymal calcifications, cortical atrophy and cerebellar hypoplasia.	SVD of live infant at 39 weeks
28	Carvalho et al., 2016 [16]	Brazil	10 weeks	Microcephaly, left ventriculomegaly, increase cisterna magna.	CS delivery of live infant at 40 weeks
28.2	Cortes et al., 2018 [14]	Colombia	NR	Enlarged subarachnoid space, decreased brain volume, ventriculomegaly, malformations in cortical development, simplified gyral pattern, hypoplasic corpus callosum	NR
29	Pomar et al., 2017 [13]	French Guiana	Second trimester	Lentostriatal vasculopathy	Delivery at 39 weeks
29	Carvalho et al., 2016 [16]	Brazil	16 weeks	Microcephaly, periventricular calcifications, megacisterna magna, cerebellar vermis hypoplasia. Thymic and liver calcifications, fetal growth restriction	Stillbirth, labour induced
29	Mlakar et al., 2016 [17]	Brazil	13 weeks	Microcephaly, parenchymal calcifications. Fetal growth restriction, placental calcifications.	TA at 32 weeks
29.2	Besnard et al., 2016 [15]	French Polynesia	NR	Microcephaly, parenchymal calcifications, placental microcalcifications, ventriculomegaly, occipital pseudo-cyst, absent corpus callosum, absent cavum septi pellucidi, enlarged pericerebral space	TA at unknown weeks
29.2	Oliveira Melo et al., 2016 [18]	Brazil	NR	Ventriculomegaly, corpus callosum not visualized, thalami not developed, thin pons and brainstem, non-homogeneous small mass seen at position of basal ganglia, lateral ventricles and fourth ventricle calcifications. Cataracts bilaterally, intraocular calcifications and one eye smaller in size than the other	Unknown
29.3	Besnard et al., 2016 [15]	French Polynesia	First trimester	Microcephaly, ventriculomegaly, occipital pseudo-cyst, enlarged pericerebral space	TA at unknown weeks
30.1	Oliveira Melo et al., 2016 [18]	Brazil	NR	Brain atrophy, calcifications in frontal lobes including caudate, lentostriatal vessels and cerebellum. Corpus callosal and vermian dysgenesis, enlarged cisterna magna.	Unknown
31	Carvalho et al., 2016 [16]	Brazil	8 weeks	Microcephaly, parenchymal and cerebellar calcifications.	SVD of live infant at 40 weeks
31	Carvalho et al., 2016 [16]	Brazil	9 weeks	Microcephaly, parenchymal and cerebellar calcifications, brain atrophy, cerebellar hypoplasia. Right clubfoot, nuchal skin wrinkling, increase cisterna magna, fetal growth restriction.	CS delivery of live infant at 39 weeks
31	Carvalho et al., 2016 [16]	Brazil	NR	Polyhydramnios, parenchymal and cerebellar calcifications. Hepatomegaly, hyperflexion of fingers, and hyperechogenicity of aortic valve, mitral valve and aortic root.	SVD of live infant at 38 weeks
31	Carvalho et al., 2016 [13]	Brazil	NR	Microcephaly, ventriculomegaly, parenchymal calcifications	CS delivery of live infant at 39 weeks
33	Cortes et al., 2018 [14]	Colombia	NR	Enlarged subarachnoid space, decreased brain volume, ventriculomegaly, dysgenetic corpus callosum	NR
34.2	Cortes et al., 2018 [14]	Colombia	NR	Enlarged subarachnoid space, decreased brain volume, ventriculomegaly, malformations in cortical development, simplified gyral pattern, dysgenetic corpus callosum	NR
35	Carvalho et al., 2016 [16]	Brazil	NR	Microcephaly, parenchymal and cerebellar calcifications, ventriculomegaly	CS delivery of live infant at 39 weeks
36	Carvalho et al., 2016 [16]	Brazil	NR	Microcephaly. Right clubfoot, fetal growth restriction	Fetal demise, CS at 38 weeks
36	Carvalho et al., 2016 [16]	Brazil	11 weeks	Microcephaly	CS delivery of live infant at 40 weeks
37	Pomar et al., 2017 [13]	French Guiana	Second trimester	Microcephaly, callosal hypoplasia, gyration anomalies	Delivery at 39 weeks
37	Carvalho et al., 2016 [16]	Brazil	8 weeks	Microcephaly, parenchymal and cerebellar calcifications, left ventriculomegaly	SVD of live infant at 39 weeks
38	Carvalho et al., 2016 [16]	Brazil	10 weeks	Microcephaly, periventricular and parenchymal calcifications, ventriculomegaly. Fetal growth restriction.	CS delivery of live infant at 39 weeks
38	Carvalho et al., 2016 [16]	Brazil	16 weeks	Microcephaly, parenchymal and periventricular calcifications, left ventriculomegaly. Fetal growth restriction, oligohydramnios	CS delivery of live infant at 39 weeks
38	Carvalho et al., 2016 [16]	Brazil	NR	Microcephaly, parenchymal and cerebellar calcifications, ventriculomegaly	SVD of live infant at 39 weeks
39	Carvalho et al., 2016 [16]	Brazil	NR	Microcephaly, parenchymal calcifications.	SVD of live infant at 39 weeks
NR	Pomar et al., 2017 [13]	French Guiana	First trimester	Microcephaly, cerebellar hypoplasia, ventriculomegaly with irregular ventricular walls, periventricular hyperechogenicity, callosal hypoplasia with calcifications, parenchymal califications	NR
NR	Pomar et al., 2017 [13]	French Guiana	Second trimester	Parenchymal hyperechogenicity	Fetal demise at 40 weeks
NR	Pomar et al., 2017 [13]	French Guiana	First trimester	Microcephaly	Fetal demise at 19 weeks

*NR* not reported, *SVD* spontaneous vaginal delivery, *CS* caesarean section, *TA* therapeutic abortion

We identified persistent viremia at 30-days post symptom onset in the context of prolonged symptomatology and documented fever. For most patients viremia is present for 3–5 days after symptom onset [3]. However, case reports have emerged demonstrating prolonged viremia in pregnant women [21]. One prospective cohort study found that clearance of ZIKV in serum, urine, and semen was seen in 95% of patients by 54 days, 39 days, and 81 days, respectively [22]. Furthermore urine testing in our patient was negative, conflicting with previous reports suggesting urine PCR is positive for longer than serum [23]. Detecting prolonged viremia may prove to be a valuable prognostic indicator for pregnant women. It is likely that viral detection in maternal serum represents high levels of fetal viral replication. Pregnant women may have greater difficulty clearing the virus, which may then lead to fetal infection. Further study investigating this finding is warranted.

The timing of fetal abnormalities ranges from identification at 14-weeks (this report) to post-natal diagnosis. Table 1 summarizes the spectrum of fetal ultrasound abnormalities detected. The spectrum of severity ranges from mild ocular or auditory abnormalities, to microcephaly, to severe brain or musculoskeletal malformations [24]. Additional information on congenital Zika syndrome is needed to better understand the spectrum of illness, however, 5 features have been recognized that are rarely seen with other congenital infections. These include severe microcephaly with a partially collapsed skull, thin cerebral cortices with subcortical calcifications, macular scarring with focal pigmentary retinal mottling, congenital contractures, and early hypertonia with extrapyramidal involvement [25].

Zika virus is currently endemic to most of South and Central America, the Caribbean, and recently areas of southern Florida and Texas in the United States. International guidelines have advised that pregnant women should avoid travel to areas with ongoing transmission of ZIKV [3]. Women unable to avoid travel are advised to use strict personal protective measures including light-coloured long-sleeved clothing, insect repellent containing 20–30% DEET or 20% icaridin, stay in accommodations with screens or air-conditioning, and permethrin treated clothes and bed nets if outdoors [3]. Sexual transmission of ZIKV has also been described, but based on our current understanding of ZIKV epidemiology, likely represents a minor contributor to disease spread. To reduce the possibility of sexual transmission, we recommend that men and women avoid unprotected sexual activity with a male partner who has returned from an endemic area within six months, pending further understanding of the duration of transmission. Recent reports have demonstrated detectable virus in semen up to 6 months from symptom onset [26]. Women are further advised to defer conception for 2 months following travel to an area of ongoing ZIKV transmission, and for 6 months if their male partner accompanied them on travel [3].

Our current approach to diagnosis of pregnant patients exposed to ZIKV is outlined in recent guidelines published by the CDC [27]. Up-to-date testing algorithms can be accessed here: https://www.cdc.gov/zika/hc-providers/testing-for-zikavirus.html. Testing all exposed pregnant women by PCR within two weeks of symptom onset or exposure is recommended. Those negative for ZIKV by PCR should be tested for ZIKV IgM and if positive, confirmed by PRNT. Pregnant women 2–12 weeks from exposure, or symptom onset, should be tested for ZIKV IgM and if positive or equivocal should be tested for ZIKV RT-PCR. As illustrated by our case, however, viremia in women carrying congenitally infected fetuses may be prolonged, and in pregnant women who continue to be symptomatic beyond 2 weeks, ZIKV PCR may be considered.

Recommendations surrounding the management of pregnant women exposed to Zika virus will continue to evolve along with our understanding of the epidemiology and pathogenesis of disease. Expert bodies continue to recommend screening all pregnant women with travel to areas with ongoing transmission of ZIKV. Positive tests should prompt further evaluation with serial ultrasound assessments and possible amniocentesis for ZIKV RT-PCR to facilitate counselling of patients.

Our report has some limitations. We identified higher levels of ZIKV in fetal neurological tissue compared to others, however, due to the dilation and curettage procedure performed, we cannot exclude potential cross-contamination of tissues.

## Conclusions

Congenital ZIKV syndrome is a newly recognized complication of this arboviral infection which has spread throughout the Americas. This case report demonstrates that pregnant women can have prolonged viremia and early ultrasounds may detect significant fetal abnormalities. Human vaccine trials are currently underway, however personal protective measures to avoid mosquito bites and deferral of travel to risk areas are currently the cornerstone of prevention, and should be advocated by front line health care providers to all pregnant patients considering travel to ZIKV endemic areas.
